# 1049. Minimal Transient HIV-1 Viremia Following Vaccination Regimens Containing AD26. ZEBOV and MVA-BN-Filo in ART-Suppressed People Living with HIV

**DOI:** 10.1093/ofid/ofab466.1243

**Published:** 2021-12-04

**Authors:** Benjamin L Custer, Betty Mwesigwa, Fredrick Sawe, Janet Oyieko, Nyanda Ntinginya, Ilesh Jani, Michael Iroezindu, Jack Hutter, Linda Jagodzinski, Georgi Shukarev, Leigh Anne Eller, Lucy Ward, Rachel Overman, Janice M Rusnak, Callie Bounds, Christopher Badorrek, Christina Polyak, Allahna L Esber, Amber D Moodley, Chi Tran, Auguste Gaddah, Kerstin Luhn, Macaya Douoguih, Cynthia Robinson, Julie A Ake

**Affiliations:** 1 Walter Reed National Military Medical Center, Rockville, Maryland; 2 Makerere university Walter Reed Project, Kampala, Kampala, Uganda; 3 Henry M. Jackson Foundation Medical Research International, Kenya; U.S. Military HIV Research Program, Walter Reed Army Institute of Research, Silver Spring, Maryland, USA, Kericho, Rift Valley, Kenya; 4 Henry M. Jackson Foundation Medical Research International, Kenya; Kenya Medical Research Institute/U.S. Army Medical Research Directorate-Africa/Kenya, Kisumu, Nyanza, Kenya; 5 National Institute for Medical Research-Mbeya Medical Research Center, Mbeya, Tanzania, Mbeya, Mbeya, Tanzania; 6 Instituto Nacional de Saúde, Maputo, Maputo, Mozambique; 7 U.S. Military HIV Research Program, Walter Reed Army Institute of Research, Silver Spring, MD, USA, Silver Spring, Maryland; 8 Clinical Trials Center, Walter Reed Army Institute of Research, Silver Spring, Maryland, USA, Silver Spring, Maryland; 9 Walter Reed Army Institute of Research, Silver Spring, Maryland; 10 Janssen Vaccines and Prevention, Leiden, Netherlands, Leiden, Zuid-Holland, Netherlands; 11 US Military HIV Research Program/HJF, Bethesda, Maryland; 12 U.S. Department of Defense (DOD) Joint Program Executive Office for Chemical, Biological, Radiological and Nuclear Defense (JPEO-CBRND), Joint Project Manager for Chemical, Biological, Radiological, and Nuclear Medical (JPM CBRN Medical), Fort Detrick, Maryland, USA, Fort Detrick, Maryland; 13 Contract Support for U.S. Department of Defense (DOD) Joint Program Executive Office for Chemical, Biological, Radiological and Nuclear Defense (JPEO-CBRND), Joint Project Manager for Chemical, Biological, Radiological, and Nuclear Medical (JPM CBRN Medical), Fort Detrick, Maryland; 14 Joint Project Manager for Chemical, Biological, Radiological, and Nuclear Medical, Fort Detrick, Maryland; 15 Contract Support for U.S. Department of Defense (DOD) Joint Program Executive Office for Chemical, Biological, Radiological and Nuclear Defense (JPEO-CBRND), Joint Project Manager for Chemical, Biological, Radiological, and Nuclear Medical (JPM CBRN Medical), Fort Detrick, Maryland, USA, Fort Detrick, Maryland; 16 The Henry M. Jackson Foundation for the Advancement of Military Medicine, Bethesda, MD and Walter Reed Army Institute of Research, Silver Spring, MD, Bethesda, Maryland; 17 U.S. Military HIV Research Program, Walter Reed Army Institute of Research, Silver Spring, Maryland; Henry M. Jackson Foundation for the Advancement of Military Medicine. Bethesda, Maryland, USA, Rockville, Maryland; 18 HJF/MHRP, Silver Spring, Maryland; 19 Janssen Research and Development, Beerse, Antwerpen, Belgium; 20 Janssen Vaccines and Prevention, Leiden, Zuid-Holland, Netherlands; 21 Walter Reed Army Institute of Research, Silver Spring, MD, Silver Spring, Maryland

## Abstract

**Background:**

Ebola Virus Disease (EVD) outbreaks primarily occur in the HIV endemic setting of Sub-Saharan Africa. Transient increases in HIV viral load (VL), or blips, have been described following routine vaccinations. We characterized VL blips among PLWH enrolled in a phase 2 trial of a heterologous two-dose EVD vaccine.

**Methods:**

In EBL2003, adult participants with and without HIV were randomized 1:4 to receive placebo or vaccine. Part A in the US studied MVA-BN-Filo followed by Ad26.ZEBOV 14 days later. Part B in Africa evaluated this MVA/Ad26 regimen and also a schedule of Ad26.ZEBOV followed by MVA-BN-Filo 29 days later. VL was assessed at screening, pre-vaccination, and 21, 42, 180, and 365 days post dose 2. Participants with VL < 20 copies/mL at the first 2 visits who received both doses and had complete VL data through 42 days post dose 2 were evaluated. Blips were defined as a post-injection VL ≥ 20 copies/mL no later than 42 days post dose 2, with subsequent return to VL < 20 copies/mL.

**Results:**

A total of 277 PLWH on antiretroviral therapy (ART) were assessed; 73.3% (203) had baseline virologic suppression, and 89.2% (181) of those received both doses with complete VL data for inclusion in the analysis. Overall, 19.9% (36) experienced blips: 20.0% (29) of vaccinees vs 19.4% (7) of placebo recipients (p=1.0). All baseline suppressed participants with post-injection viremia subsequently regained suppression. Among vaccinees, the mean blip VL was 192 copies/mL, and the mean blip duration was 56 days, which was not significantly different from placebo. Of all blips, only 2 were > 1,000 copies/mL. Blips occurred in 24.0% (25) of Ad26/MVA recipients, and 9.7% (4) of MVA/Ad26 recipients (p=0.07). A dose of Ad26 was associated with a blip in 6.9% (10) of recipients vs 13.1% (19) for MVA recipients (p=0.12). Regardless of regimen, dose 1 was associated with a blip in 8.3% (12) of vaccinees, compared to 11.7% (17) of vaccinees for dose 2 (p=0.43).

**Conclusion:**

Among successfully treated PLWH, we observed low magnitude post-dose HIV blips that were not more common in vaccine vs. placebo recipients and did not result in loss of virologic suppression. This data is favorable for the deployment of the EVD vaccines in this trial in areas of high HIV endemicity.

**Disclosures:**

**Benjamin L. Custer, M.D.**, **Alexion Pharmaceuticals** (Shareholder)**Armata Pharmaceuticals** (Shareholder)**Biomarin Pharmaceutical** (Shareholder)**Crispr Therapeutics** (Shareholder)**CVS Health Corp** (Shareholder)**Editas Medicine** (Shareholder)**Gilead** (Shareholder)**Glaxo Smith Kline** (Shareholder)**Hologic Inc** (Shareholder)**Merck** (Shareholder)**Mesoblast LTD** (Shareholder)**Pfizer** (Shareholder)**Sanofi** (Shareholder)**Unitedhealth Group** (Shareholder)**Vertex Pharmaceuticals** (Shareholder) **Georgi Shukarev, MD**, **Janssen** (Employee) **Auguste Gaddah, PhD**, **Janssen Pharmaceutica N.V** (Employee) **Kerstin Luhn, PhD**, **Janssen Vaccines and Prevention** (Employee, Shareholder) **Macaya Douoguih, MD, MPH**, **Janssen** (Employee) **Cynthia Robinson, MD**, **Janssen Vaccines** (Employee)

